# SB-334867 (an Orexin-1 Receptor Antagonist) Effects on Morphine-Induced Sensitization in Mice—a View on Receptor Mechanisms

**DOI:** 10.1007/s12035-018-0993-0

**Published:** 2018-03-20

**Authors:** Małgorzata Łupina, Maciej Tarnowski, Irena Baranowska-Bosiacka, Sylwia Talarek, Piotr Listos, Jolanta Kotlińska, Izabela Gutowska, Joanna Listos

**Affiliations:** 1 Department of Pharmacology and Pharmacodynamics, Medical University of Lublin, Chodźki 4a St., 20-093 Lublin, Poland; 2 Department of Physiology, Pomeranian Medical University, Powstańców Wlkp. 72 Av., 70-111 Szczecin, Poland; 3 Department of Biochemistry and Medical Chemistry, Pomeranian Medical University, Powstańców Wlkp. 72 Av., 70-111 Szczecin, Poland; 4 Department and Clinic of Animal Internal Diseases, Sub-Department of Pathomorphology and Forensic Medicine, Faculty of Veterinary Medicine, University of Life Sciences in Lublin, 30 Av, 20-612 Lublin, Poland; 5 Department of Biochemistry and Human Nutrition, Pomeranian Medical University, Broniewskiego 24 Str., 71-460 Szczecin, Poland

**Keywords:** Opioid addiction, Dopamine receptor expression, Adenosine receptor expression, GFAP, Iba-1

## Abstract

The present study focused upon the role of SB-334867, an orexin-1 receptor antagonist, in the acquisition of morphine-induced sensitization to locomotor activity in mice. Behavioral sensitization is an enhanced systemic reaction to the same dose of an addictive substance, which assumingly increases both the desire for the drug and the risk of relapse to addiction. Morphine-induced sensitization in mice was achieved by sporadic doses (five injections every 3 days) of morphine (10 mg/kg, i.p.), while a challenge dose of morphine (10 mg/kg) was injected 7 days later. In order to assess the impact of orexin system blockade on the acquisition of sensitization, SB-334867 was administered before each morphine injection, except the morphine challenge dose. The locomotor activity test was performed on each day of morphine administration. Brain structures (striatum, hippocampus, and prefrontal cortex) were collected after behavioral tests for molecular experiments in which mRNA expression of orexin, dopamine, and adenosine receptors was explored by the qRT-PCR technique. Additionally, the mRNA expression of markers, such as GFAP and Iba-1, was also analyzed by the same technique. SB-334867 inhibited the acquisition of morphine-induced sensitization to locomotor activity of mice. Significant alterations were observed in mRNA expression of orexin, dopamine, and adenosine receptors and in the expression of GFAP and Iba-1, showing a broad range of interactions in the mesolimbic system among orexin, dopamine, adenosine, and glial cells during behavioral sensitization. Summing up, the orexin system may be an effective measure to inhibit morphine-induced behavioral sensitization.

## Introduction

Morphine is a valuable drug in clinical practice for its analgesic efficacy. Its use is, however, limited because of addictive properties. Morphine acts on *μ* opioid receptors in the ventral tegmental area (VTA) and inhibits the γ-aminobutyric acid (GABA) system, increasing dopamine release and inducing euphoria for the user [1]. It is well-known that a chronic administration of morphine leads to physical and psychological addiction [2, 3]. An intermittent administration of that drug develops behavioral sensitization, defined as an enhanced systemic reaction to the same dose of morphine or any other addictive substance [4, 5]. Behavioral sensitization is a long-lasting phenomenon, associated with both context-dependent and context-independent factors which may lead to addiction relapse [6, 7]. Sensitization is commonly manifested in behavioral experiments by increased locomotor activity of participating animals (sensitization to the locomotor activity), promoting a greater desire for the drug or craving; it may also increase the risk of relapse to former drug addiction [8]. Although the mechanisms, which are involved in behavioral sensitization, are not yet fully understood, an influence of the mesocorticolimbic system in that phenomenon has been repeatedly documented [7, 9]. In general, the mesocorticolimbic pathway is referred to as a dopaminergic projection, derived from VTA into the nucleus accumbens and the prefrontal cortex [10]. VTA is thought to play a predominant role in sensitization development by releasing dopamine into the forebrain [11]. An increased dopamine neurotransmission was observed during sensitization development [12]. In opioid-induced behavioral sensitization, the influence of various neurotransmitters and neuromodulators was experimentally demonstrated, including dopamine [12], glutamate [9], serotonin [13, 14], adenosine [15, 16], nitric oxide [17], and others. Although the mechanisms of behavioral sensitization have often been evaluated, no effective pharmacological treatment options have thus far been identified to be able to reduce that phenomenon.

The orexin system seems to be a promising strategy, as orexin neurons send extensive projections throughout the central nervous system (CNS) and exert comprehensive pharmacological effects. Orexins evoke the effects via two metabotropic receptors: orexin type 1 (OX-1) and orexin type 2 (OX-2) which are widely distributed in the cortex, the hypothalamus, the thalamus, and other brain areas [18–20]. Orexins are involved in sleep regulation and wakefulness, arousal, feeding, endocrine activity, visceral functions, energy homeostasis, and drug addiction [21–23]. There were some suggestions that OX-1 receptors were more involved in drug-seeking behavior [24, 25], and OX-2 receptors were strongly involved in the regulation of sleep and arousal [26]. Novel data presents some involvement of both subtypes of orexin receptors in the development of drug-seeking behaviors [27]. Several experiments confirmed the orexin system involvement in the activity of addictive drugs. For example, terminals of lateral hypothalamus orexin neurons are connected with dopaminergic neurons in VTA [28]—a cerebral area which is strongly involved in behavioral sensitization. Moreover, SB-334867, the OX-1 receptor antagonist, reduced ethanol consumption by high-ethanol-preferring rats [29] and the expression of ethanol-induced sensitization of mice [30]. It also inhibited the stress-induced [31] and the environment-induced [32] cocaine-seeking behavior. In another study, SB-334867 suppressed the acquisition, but not the expression, of cocaine-induced sensitization to the locomotor activity in rats [28]. On the other hand, the blockade of both orexin receptors by almorexant did not affect the expression of that sensitization [33]. Quarta et al. [34] and Winrow et al. [35] demonstrated that SB-334867, a selective antagonist, and DORA, a non-selective antagonist, respectively, reduced the expression of amphetamine-induced sensitization to the locomotor activity in rats and mice. An increasing number of literature reports present the orexin system to be involved in morphine dependence. It was indicated that SB-334867 decreased morphine withdrawal signs [36] and reduced the rewarding effect of morphine in the conditioned place preference test [37]. Some novel data on SB-334867 demonstrated that when it was microinjected into the cerebral ventricle, it reduced naloxone-induced elevation of the cAMP level in locus coeruleus neurons [38]. All those results confirm the important role of the orexin system in the addiction mechanism, and it also seems that a pharmacological modification of the orexin pathway may prove a valuable method in the future to effectively inhibit that phenomenon.

The present study investigated the scope and character of the orexin system involvement in the acquisition of morphine-induced sensitization to locomotor activity in mice. In the first step, SB-334867 was applied in behavioral experiments. That selective OX-1 receptor antagonist showed at least 50-fold selectivity for OX-1 receptor over the OX-2 receptor [39–41]. After the behavioral study, molecular experiments were performed in the following three brain areas: the striatum, the hippocampus, and the prefrontal cortex. In molecular experiments, the mRNA expression of orexin, dopamine, and adenosine receptors was investigated by the real-time quantitative reverse-transcription polymerase chain reaction (qRT-PCR technique). The objectives of those experiments included the relationships, observed in mesolimbic brain areas in result of sporadic SB-334867 administration in morphine-sensitized mice. In our investigations, we concentrated on the expression of dopamine receptors because of the crucial role of dopamine in behavioral sensitization [9, 12, 16, 42]. We also intended to recognize the expression type of both orexin receptors in the studied sensitization. Our subsequent experiments aimed to assess the expression of adenosine receptors, observed in that phenomenon, taking into account the close relationships between adenosine and dopamine receptors in the mesolimbic system [43]. The latest data suggest that a long-term administration of addictive substances may induce a neuroinflammatory process in the brain [44, 45]. Neuroinflammation is often approached in preclinical experiments as a kind of a changed expression of the markers of astrogliosis (glial fibrillary acidic protein (GFAP)) and microgliosis (ionized calcium-binding adapter molecule 1 (Iba-1)). It was evidenced that a long-term administration of cocaine [46, 47] or metamphetamine [46, 48] produced neuroinflammation, manifested by higher expressions of GFAP and Iba-1; however, the mechanisms of morphine-induced neuroinflammation are not still confirmed [49]. Therefore, we undertook to analyze the mRNA expression of astrogliosis (GFAP) and microgliosis (Iba-1) markers in the same brain areas (the striatum, the hippocampus, and the prefrontal cortex). Our study shows how significant the orexin system is in the acquisition phenomenon of morphine-induced sensitization and presents a broad spectrum of neural and glial relationships which may be involved in that process.

## Materials and Methods

### Animals

The experiments were performed on male Swiss mice (20–30 g). The animals were fed a standard pelleted diet of Murigran (Bacutil, Motycz) and water ad libitum. During the experiments, six to eight animals were kept per cage at room temperature of 22 ± 1 °C and exposed to a normal day/night cycle. All the experiments were carried out between 8:00 a.m. and 3:00 p.m. The animals were handled once a day for 7 days preceding the experiments. The study was performed, according to the National Institute of Health Guidelines for the Care and Use of Laboratory Animals and the European Community Council Directive for Care and Use of Laboratory Animals, and was approved by the Local Ethics Committee (The Medical University of Lublin Committee on the Use and Care of Animals).

### Drugs in Behavioral Experiments

The following drugs were used in the experiments: morphine hydrochloride trihydrate (Cosmetic Pharma, Poland) and 1-(2-methyylbenzoxanzol-6-yl)-3-[1,5]naphthyridin-4-yl-urea hydrochloride (SB-334867)—a selective OX-1 receptor antagonist (Tocris, UK). Morphine was dissolved in 0.9% saline, and SB-334867 was dissolved in three drops of DMSO and diluted in 0.9% saline (final the DMSO concentration 0.1%). All used substances were delivered intraperitoneally (i.p.) in a volume of 10 ml/kg. Morphine was used at the dose of 10 mg/kg, and SB-334867 was injected at the dose of 20 mg/kg. As literature data show, the minimal effective dose of SB-334867 is 30 mg/kg [24, 50–52]. According to the generally accepted principles of behavioral sensitization research, an ineffective dose of pharmacological agents (SB-334867 in our study) is recommended. Therefore, based on the literature data and on our preliminary unpublished results, the dose of SB-334867 (20 mg/kg), administered in the reported study, was subthreshold. The animals in a control group received the same volume of saline at the respective time point before the test.

### Apparatus

The locomotor activity in mice was measured in round actometer cages (32 cm in diameter, KBA 300 L, Lublin, Poland), which were placed in a sound-attenuated experimental room. Those cages were provided with two perpendicular beams of light. Each interruption of beams, caused by a moving mouse, was registered by counters as a single movement. Each mouse was placed in the same actometer. Locomotor activity was measured for the total period of 60 min.

### Procedure of Behavioral Sensitization

#### The Influence of SB-334867 (OX-1 Receptor Antagonist, 20 mg/kg, i.p.) on the Acquisition of Morphine Sensitization to Locomotor Activity

The induced morphine behavioral sensitization in mice was based on the method, described by Kuribara [53], with a modification of Kotlińska and Bocheński [54]. The animals received five injections of morphine (i.p.) at the dose of 10 mg/kg every 3 days (on the 1st, 4th, 7th, 10th, and 13th day of the experiment). Seven days after the last morphine injection (on the 20th day of the study), the mice were administered with a challenge dose of morphine (10 mg/kg, i.p.). Aiming to grade the development of behavioral sensitization, the mice were immediately placed into the actometer to record their locomotor activity for the period of 60 min. The control animals were administered with saline (i.p.).

Afterwards, the effects of SB-334867 (the selective OX-1 receptor antagonist) on the acquisition of morphine-induced sensitization were explored. SB-334867 was administered 15 min before morphine injection on the 1st, 4th, 7th, 10th, and 13th day of the experiment, but not on the 20th day. The control animals were administered with saline (i.p.).

### Procedure of Molecular Analysis

#### Analysis of mRNA Expression by the Quantitative Real-Time PCR Technique (qRT-PCR) in the Striatum, the Hippocampus, and the Prefrontal Cortex

A quantitative assessment of mRNA levels was performed by the real-time RT-PCR technique, using an ABI 7500Fast instrument with Power SYBR Green PCR Master Mix reagent. Real-time conditions were as follows: 95 °C (15 s), 40 cycles at 95 °C (15 s), and 60 °C (1 min). According to melting point analysis, only one PCR product was amplified under those conditions. Each sample was analyzed in two technical replicates, and the mean Ct values were used for further analysis. The relative target quantity, normalized to the endogenous control (*Gapdh*) gene and relative to a calibrator, is expressed as 2^−∆∆Ct^ (-fold difference), where Ct is the threshold cycle, ∆Ct = (Ct of target genes) − (Ct of endogenous control gene), and ∆∆Ct = (∆Ct of samples for target gene) − (∆Ct of calibrator for the target gene). The following primer pairs were used: *Gapdh*: 5′ - GGA GAA ACC TGC CAA GTA TGA TG -3′ and 5′ - GAC AAC CTG GTC CTC AGT GTA GC - 3′; for OX-1 receptor: 5′ - GTT ATC TGC CCA TCA GTG TCC TC -3′ and 5′ - GGT GAA GCA GGC GTA GAC G -3′; for OX-2 receptor: 5′ - GGC TTA TCT CCA AAT ATT CCG TAA -3′ and 5′ - CTC TGA ACC ACA GAA GAA GTT CC -3; for D1 receptor: 5′- GTA GCC ATT ATG ATC GTC AC -3′ and 5′- GAT CAC AGA CAG TGT CTT CAG-3′; for D2 receptor: 5′- TGA CAG TCC TGC CAA ACC AGA GAA -3′ and 5′- TGG GCA TGG TCT GGA TCT CAA AGA -3′; for A1 receptor: 5′- ACA AAA ACC AGT GGT GGA GTG A-3′ and 5′- TCT GTC CCC TCC CCT TGT C- 3′; for A2A receptor: 5′- TGG CTT GGT GAC GGG TAT G -3′ and 5′- CGC AGG TCT TTG TGG AGT TC -3′; for Iba-1: 5′- GAT TTG CAG GGA GGA AAA GCT -3′ and 5′- AAC CCC AAG TTT CTC CAG CAT -3′; for GFAP: 5′- TCC TGG AAC AGC AAA ACA AG-3′ and 5′- CAG CCT CAG GTT GGT TTC AT-3′.

### Statistical Analysis

The obtained results are presented in figures as mean values ± SD for behavioral and molecular experiments. They were statistically calculated by the GraphPad Prism Software package (version 5.04), using a two-way (for behavioral experiment) and one-way (for molecular experiments) analysis of variance (ANOVA). Post hoc comparisons were carried out by the Tukey’s test. A probability (*P*) value below 0.05 (*P* < 0.05) was considered as statistically significant. Each group of the animals in the behavioral experiment included 8–12 mice, and the number of samples in molecular experiments was 6–8.

## Results

### Influence of SB-334867 (the OX-1 Receptor Antagonist, 20 mg/kg, i.p.) on the Acquisition of Morphine Induced Sensitization to Locomotor Activity

**Fig. 1:**
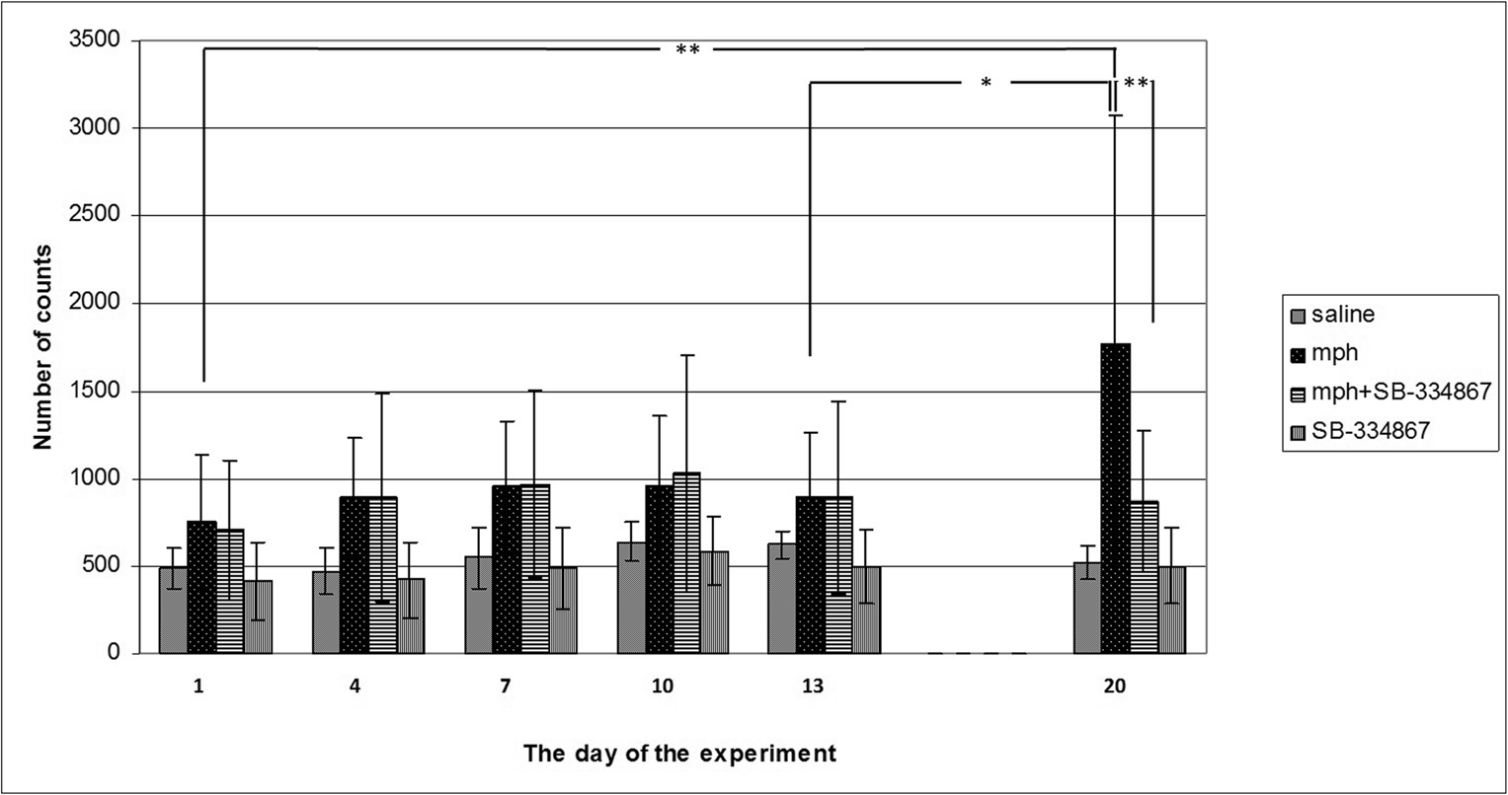
Influence of SB-334867 (20 mg/kg, i.p.) on the acquisition of morphine-induced sensitization to locomotor activity. Experimental mice received five injections of morphine (10 mg/kg, i.p.) on the 1st, 4th, 7th, 10th, and 13th to develop sensitization and a subsequent injection of morphine (challenge dose, 10 mg/kg, i.p.) was done on the 20th day (mph-treated group). SB-334867 (20 mg/kg, i.p.) was administered 15 min before each morphine injection but not before the morphine challenge dose (mph + SB-334867). After each morphine injection, locomotor activity was measured for 60 min. The presented data represent means ± SD and are expressed as the number of counts. *n* = 8–12 mice per group; *P < 0.05, **P < 0.01 (Tukey’s test)

An intermittent administration of morphine (10 mg/kg) gradually potentiated the locomotor activity of mice. A two-way ANOVA showed a significant effect of the drug (*F*_1,36_ = 11.10, *P* = 0.0015), day (*F*_2,36_ = 3.177, *P* = 0.0493), and interaction (*F*_2,36_ = 3.54, *P* = 0.00357). Post hoc comparisons were carried out by the Tukey’s test, demonstrating significant effects of a challenge dose of morphine on the 20th day of the study, in comparison with the 1st and the 13th day (*P* < 0.01 and *P* < 0.05, respectively) (see Fig. 1).

A concomitant administration of morphine and SB-334867 significantly decreased the effect of the morphine challenge dose on the 20th day in mice in comparison with the sporadically morphine-treated group (*P* < 0.01) (Fig. 1).

Moreover, post hoc comparisons by the Tukey’s test showed no significant locomotor activity changes between the saline- and morphine-treated group on the first day of the experiment. Similarly, neither SB-334867 alone nor SB-334867 with morphine had any influence on the locomotor activity of the studied animals. On the same day, SB-334867 (20 mg/kg) did not affect locomotor activity in the animals, measured during the whole experiment (see Fig. 1).

### Influence of SB-334867 on the Acquisition of Morphine-Induced Sensitization to Locomotor Activity. The Role of mRNA OX-1 and OX-2 Receptor Expression in Studied Brain Structures

**Fig. 2:**
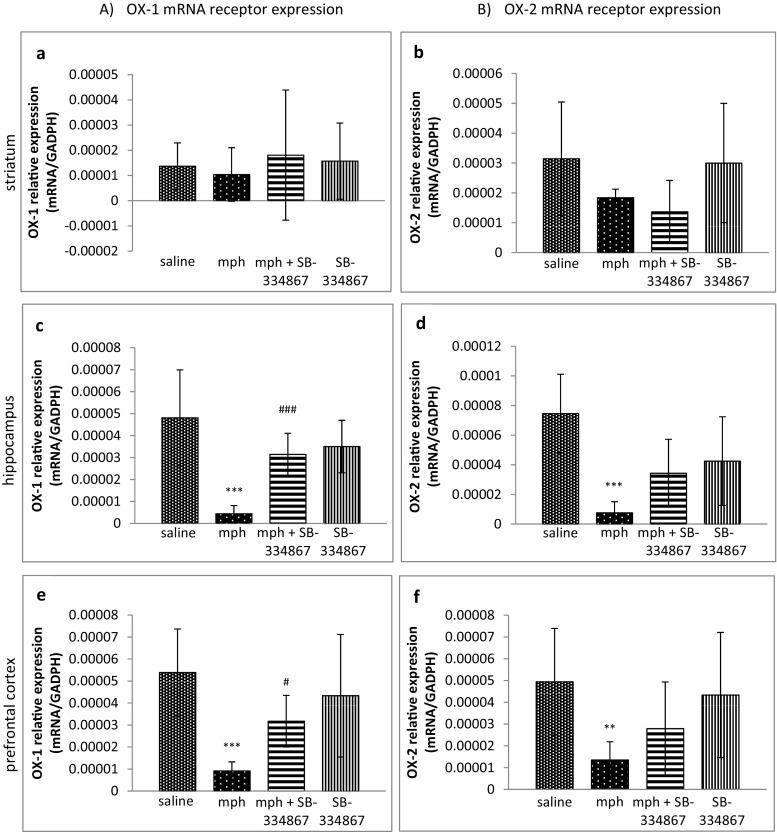
Influence of SB-334867 on the acquisition of morphine-induced sensitization to locomotor activity. The role of mRNA OX-1 (**a**, **c**, **e**) and OX-2 (**b**, **d**, **f**) receptor expression in the striatum, the hippocampus, and the prefrontal cortex. The results are expressed as means ± SD (*n* = 6–8 samples); **P < 0.01, ***P < 0.001 vs. the saline-administered group; #*P* < 0.05, ###*P* < 0.001 vs. the mph-treated group (Tukey’s test)

There were no significant differences between OX-1 and OX-2 mRNA receptor expression levels in the striatum (see Fig. 2a, b).

Regarding the hippocampus and the prefrontal cortex, the one-way ANOVA analysis revealed significant changes in mRNA expression of OX-1 receptors in mice (the hippocampus *F*_3,29_ = 15.60, *P* < 0.0001; the prefrontal cortex *F*_3,29_ = 8.727, *P* = 0.0003). Post hoc comparisons by the Tukey’s test demonstrated a significant decrease (*P* < 0.001) in mRNA expression of OX-1 receptors in both structures in the morphine-treated groups, in comparison with the saline-treated group, and that SB-334867 reversed that decrease (the hippocampus *P* < 0.001; the prefrontal cortex *P* < 0.05) (Fig. 2c, e).

The one-way ANOVA analysis showed significant changes in OX-2 receptor mRNA expression in the hippocampus and in the prefrontal cortex (the hippocampus *F*_3,29_ = 2.985, *P* = 0.0474; the prefrontal cortex *F*_3,29_ = 5.062, *P* = 0.0061). Post hoc comparisons by the Tukey’s test demonstrated a significant decrease (the hippocampus *P* < 0.001; the prefrontal cortex *P* < 0.01) in OX-2 receptor mRNA expression in the morphine-treated group, compared to the saline-treated mice. SB-334867 did not reverse the reduction, either in the hippocampus or in the prefrontal cortex (see Fig. 2d, f).

### Influence of SB-334867 on the Acquisition of Morphine-Induced Locomotor Activity Sensitization. The Role of D1 and D2 Receptor mRNA Expressions in Studied Brain Structures

**Fig. 3:**
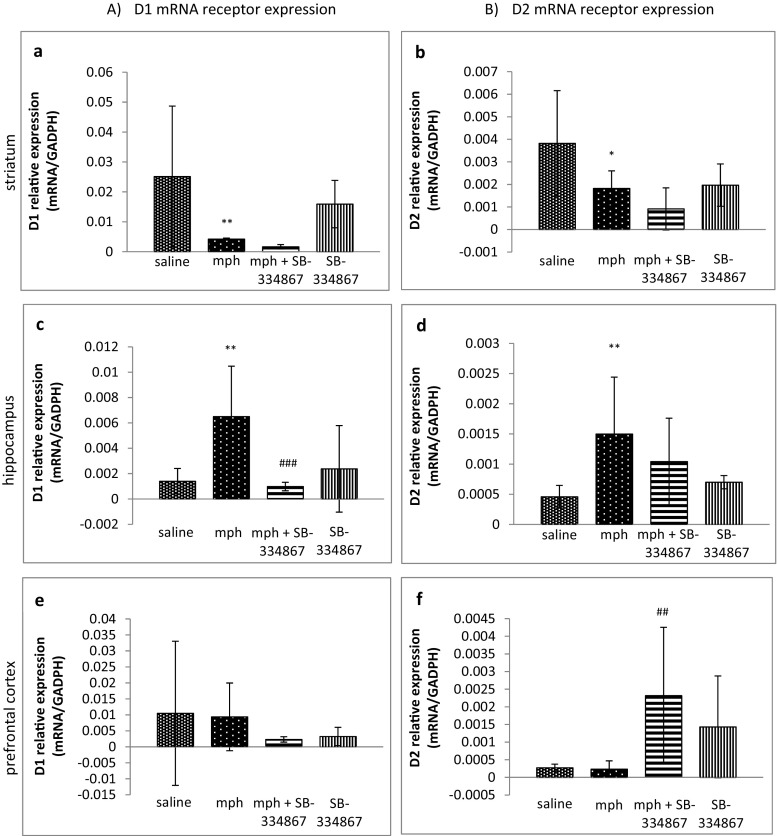
Influence of SB-334867 on acquisition of morphine-induced sensitization to locomotor activity. The role of mRNA D1 (**a**, **c**, **e**) and D2 (**b**, **d**, **f**) receptor expression in the striatum, the hippocampus, and the prefrontal cortex. The results are expressed as means ± SD (*n* = 6–8 samples); *P < 0.05, **P < 0.01 vs. the saline-administered group; ###*P* < 0.001 vs. mph-treated group (Tukey’s test)

A one-way ANOVA analysis revealed significant changes in D1 and D2 receptor mRNA expression in the striatum of the mice (*F*_3,29_ = 9.101, *P* = 0.0002). Post hoc comparisons by the Tukey’s test demonstrated significantly lower mRNA D1 and D2 receptor expression levels (*P* < 0.01 and *P* < 0.05, respectively) in the morphine-treated group than in the control mice (see Fig. 3a, b).

The one-way ANOVA analysis showed significant changes in D1 and D2 receptor mRNA expression in the hippocampus of the mice (*F*_3,29_ = 7.663, *P* = 0.0006; *F*_3,30_ = 4.782, *P* = 0.0077, respectively). Post hoc comparisons by the Tukey’s test demonstrated a significant increase (*P* < 0.01 for both receptors) in D1 and D2 receptor mRNA expressions in the morphine-treated mice, in comparison with the saline-treated group. SB-334867 reduced that high expression of D1 receptor (*P* < 0.001) (Fig. 3c) in the hippocampus, while SB-334867 not inducing any effect on D2 receptor expression (Fig. 3d).

Regarding the prefrontal cortex, the one-way ANOVA analysis confirmed significant changes only in D2 receptor mRNA expression (*F*_3,28_ = 5.480, *P* = 0.0043) but not in mRNA of D1 receptors. Although morphine did not induce any effect on D2 receptor mRNA expression levels, post hoc comparisons by the Tukey’s test demonstrated a significant increase (*P* < 0.01) in D2 receptor mRNA expression in the mice treated with morphine and SB-334867, compared to the morphine-treated group (see Fig. 3f). No significant changes were observed in D1 receptor expression levels in the prefrontal cortex (see Fig. 3e).

### Influence of SB-334867 on the Acquisition of Morphine-Induced Sensitization to Locomotor Activity. The Role of mRNA A1 and A2A Receptor Expression in Studied Brain Structures

**Fig. 4:**
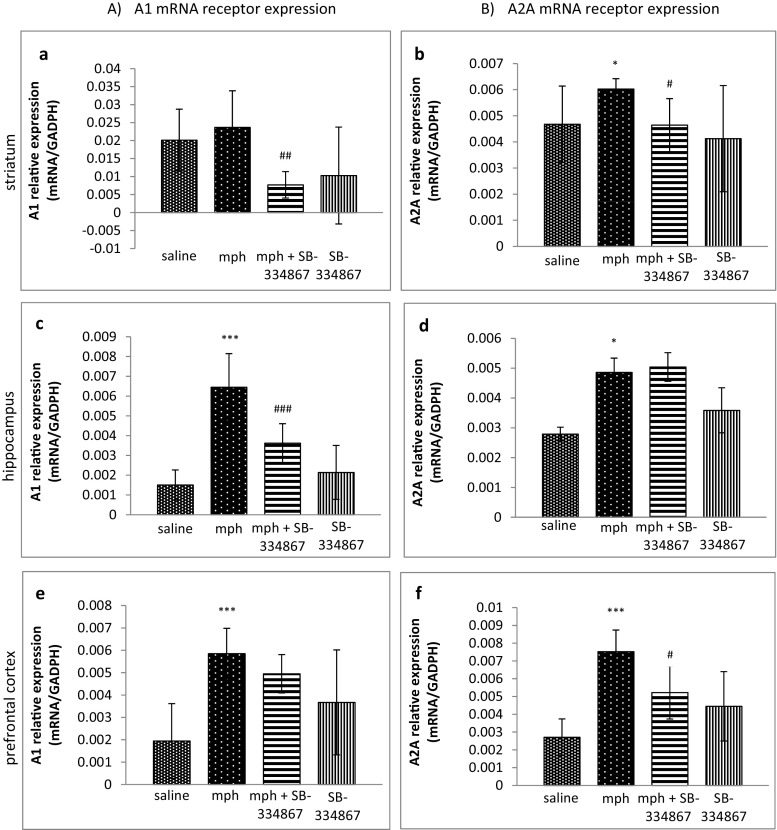
Influence of SB-334867 on acquisition of morphine-induced sensitization to locomotor activity. The role of mRNA A1 (**a**, **c**, **e**) and A2A (**b**, **d**, **f**) receptor expression in the striatum, the hippocampus, and the prefrontal cortex. The results are expressed as means ± SD (*n* = 6–8 samples); *P < 0.05, ***P < 0.001 vs. the saline-administered group; #*P* < 0.05, ##*P* < 0.01, ###*P* < 0.001 vs. mph-treated group (Tukey’s test)

The one-way ANOVA analysis revealed significant changes in A1 and A2A receptor expression levels in the striatum (*F*_3,30_ = 6.680, *P* = 0.0014; *F*_3,30_ = 4.293, *P* = 0.0107, respectively). Post hoc comparisons by the Tukey’s test demonstrated that sporadic morphine administration increased A2A receptor mRNA expression (*P* < 0.05), in comparison with the saline-treated mice (see Fig. 4b), but there were no significant alterations in A1 receptor mRNA expression in the striatum in the same group, compared to the control mice (Fig. 4a). However, a concomitant administration of morphine and SB-334867 induced a significant decrease in A1 (*P* < 0.01) and A2A (*P* < 0.05) receptor mRNA expression, in comparison with the morphine-treated mice (see Fig. 4a, b).

The one-way ANOVA analysis, performed in the hippocampus and the prefrontal cortex, revealed significant changes in adenosine receptor mRNA expression (in the hippocampus for A1 receptors *F*_3,31_ = 25.09, *P* < 0.0001; in the hippocampus for A2A receptors *F*_3,32_ = 6.650, *P* = 0.0012; in the prefrontal cortex for A1 receptors *F*_3,30_ = 10.67, *P* < 0.0001; in the prefrontal cortex for A2A receptors *F*_3,30_ = 12.76, *P* < 0.0001). The Tukey’s test showed that a sporadic administration of morphine induced higher expression levels of A1 and A2A receptor expression in the hippocampus (*P* < 0.001 and *P* < 0.05, respectively) (Fig. 4c, d) and in the prefrontal cortex (*P* < 0.001 for both receptors) (see Fig. 4e, f). SB-334867 produced a significant reduction (*P* < 0.001) of A1 receptor mRNA expression in the hippocampus (Fig. 4c) and a significant reduction (*P* < 0.05) of A2A receptor mRNA expression in the prefrontal cortex (see Fig. 4f).

### Influence of SB-334867 on the Acquisition of Morphine-Induced Sensitization to Locomotor Activity. The Role of Astrocyte and Microglial Expression in the Studied Brain Structures

**Fig. 5:**
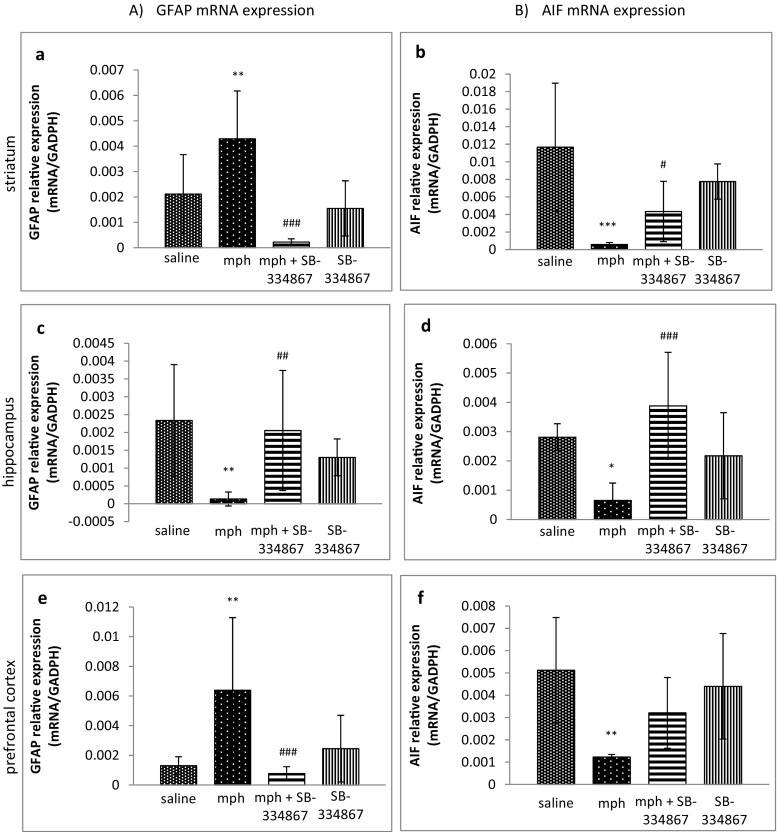
Influence of SB-334867 on acquisition of morphine-induced sensitization to locomotor activity. The role of mRNA levels of GFAP (**a**, **c**, **e**) and Iba-1 (**b**, **d**, **f**) receptor expression in the striatum, the hippocampus, and the prefrontal cortex. The results are expressed as means ± SD (*n* = 6–8 samples); *P < 0.05, **P < 0.01, ***P < 0.001 vs. the saline-administered group; #*P* < 0.05, ##*P* < 0.01, ###*P* < 0.001 vs. mph-treated group (Tukey’s test)

The one-way ANOVA analysis, carried out in all the studied brain structures, revealed significant changes in GFAP mRNA expression (the striatum *F*_3,32_ = 14.12, *P* < 0.0001; the hippocampus *F*_3,31_ = 6.169, *P* = 0.0021; the prefrontal cortex *F*_3,31_ = 7.546, *P* = 0.0006). Post hoc comparisons, carried out by the Tukey’s test in the striatum and in the prefrontal cortex, demonstrated a significant increase in GFAP mRNA expression in the morphine-treated group, compared to the control group (*P* < 0.01, for both structures). SB-334867 reversed that increase in those brain structures (*P* < 0.001, for both structures) (see Fig. 5a, e).

Opposite effects were observed in the hippocampus. The Tukey’s test showed a significant decrease in GFAP mRNA expression in the morphine-administered mice, compared to the saline-administered mice (*P* < 0.01), and that effect was reversed by SB-334867 (*P* < 0.01) (see Fig. 5c).

The one-way ANOVA analysis revealed significant changes in Iba-1 mRNA expression in all the studied brain areas (the striatum *F*_3,32_ = 17.59, *P* < 0.0001; the hippocampus *F*_3,28_ = 9.144, *P* = 0.0002; the prefrontal cortex *F*_3,31_ = 7.283, *P* = 0.0008). The Tukey’s test demonstrated that sporadic administration of morphine significantly reduced Iba-1 mRNA expression, compared to the control group in all the considered structures (the striatum *P* < 0.001, the hippocampus *P* < 0.05, the prefrontal cortex *P* < 0.01). SB-334867 reversed the morphine effect both in the striatum (*P* < 0.05) and in the hippocampus (*P* < 0.001) (see Fig. 5b, d, f).

## Discussion

The presented study aimed to investigate the impact of the orexin system on the acquisition of morphine-induced behavioral sensitization. It confirmed that SB-334867, the selective antagonist of the OX-1 receptor, inhibited the acquisition of morphine-induced sensitization to the locomotor activity in mice. The locomotor activity test is a generally accepted tool for behavioral sensitization investigation in animals [28, 34].

Current literature data on the significance of orexin receptors in morphine effects are diversified. Li et al. [55] demonstrated that a microinjection of orexin into the paraventricular nucleus of the midline thalamus inhibited locomotor activity in morphine-sensitized rats. On the other hand, Sharf et al. [52] showed, on an example of orexin knock-out mice, that orexins were not required for locomotor response to acute and chronic morphine but that OX-1 receptors could be involved in morphine-seeking behavior in wild-type mice. Later, Steiner et al. [33] demonstrated that a blockade of both orexin receptors by almorexant, non-selective drug, led to decreased expression of morphine-induced sensitization to the locomotor activity in rats. Our results confirm the knowledge on the involvement of OX-1 receptors in morphine-induced behavioral sensitization.

In the second step of the study, in order to recognize the mechanisms, involved in SB-334867-induced inhibition of morphine sensitization, molecular experiments were performed in three mesolimbic brain areas: the striatum, the hippocampus, and the prefrontal cortex. We analyzed the mRNA receptor expression of important addiction state modulators, such as orexin, dopamine, and adenosine.

In that part of the study, the significance of OX-1 and OX-2 receptors was demonstrated mainly in the hippocampus and in the prefrontal cortex. Sporadic administration of morphine significantly reduced mRNA expression of both orexin receptors in those areas, showing the involvement of both receptor types in morphine-induced sensitization. It provided evidence for the current knowledge on the role of both OX-1 and OX-2 receptors in drug-seeking behaviors [27]. SB-334867, an antagonist of OX-1 receptors, reversed the reduced expression of OX-1 receptors. In the case of OX-2 receptors, SB-334867 produced a weaker effect, probably because of the lower affinity of SB-334867 to OX-2 receptors. Although orexin receptors are localized in nucleus accumbens [56], we did not observe any changes in mRNA expression of either OX-1 or OX-2 receptor in the striatum in the morphine-sensitized mice. Neither did the administration of SB-334867 produce any effect in those animals. The striatum is a brain area which receives many inputs from various mesolimbic structures, and some interactions in that structure could be responsible in our study for the attenuation of changes in OX-1 and OX-2 receptor mRNA expression.

Some neuroadaptative changes were observed in dopamine D1 and D2 receptors in the studied animals. A significant decrease of mRNA D1 and D2 receptor expression levels was observed in the striatum of the morphine-sensitized mice. It could be caused by an increased concentration of the striatal dopamine concentration, induced by the challenge dose of morphine [9, 12]. Similarly, a reduction of D1 and D2 receptor mRNA expression was observed in that brain area of morphine-sensitized rats in our previous study, in which morphine-induced sensitization to morphine withdrawal signs was investigated in a rat model [16], as well as it may be found in studies of other authors [42].

The administration of SB-334867 in morphine-sensitized mice in the present study did not markedly influence either striatal D1 or D2 receptor mRNA expression. On the other hand, similarly to our previous study [16], a sporadic treatment with morphine induced a significant increase in D1 and D2 receptor mRNA expression in the hippocampus, showing an important role of the hippocampal dopamine receptors in morphine-induced sensitization. The selective antagonist of OX-1 receptors completely reduced D1, but not D2, receptor expression in the hippocampus, suggesting hippocampal interactions between OX-1 and D1 receptors. In the prefrontal cortex, according to our previous experiment [16], there were no significant changes in either D1 or D2 receptor expressions in morphine-sensitized mice, while a sporadic administration of SB-334867 significantly increased the prefrontal D2 receptor mRNA expression in morphine-sensitized mice. In the case of D1 receptors, a clear tendency was observed towards a decrease of D1 receptor mRNA expression, but the results were not significant, probably because of high SD value in the results. A growing number of evidences have shown that the orexin system is able to regulate the dopaminergic functions via interactions between these receptors in the VTA, where high densities of both dopamine and orexin receptors are observed [57]. For example, an administration of orexins increased firing of dopamine neurons in the VTA [58], while a blockade of OX-1 receptors reduced dopaminergic activation [59]. The exact mechanisms, underlying the connections between orexin and dopamine receptors, are still not fully understood. It seems that they not only depend on the direct inhibition of dopaminergic neurons by orexins in VTA. They are probably associated with the effects of orexins on (1) glutamatergic excitation of dopaminergic neurons in VTA, (2) the activity of dopaminergic neurons in VTA, and (3) the activation of dopamine D2 autoreceptors in VTA, which modulates the dopamine transporter activity [60]. The VTA sends many projections to other brain areas; therefore, orexin may modulate the activity of neurotransmitters in various brain structures. The obtained results suggest the existence of interactions between OX-1 and dopamine receptors in the hippocampus (D1) and in the prefrontal cortex (D2). The interactions between OX-1 and D2 receptors in the hippocampus and between OX-1 and D1 in the prefrontal cortex seem to be also possible, but our results were less expressed.

Moreover, we demonstrated in the present study an association between orexin and adenosine receptors. In the striatum, a sporadic administration of morphine induced a higher expression of A2A receptors, while SB-334867 significantly reduced striatal A1 and A2A receptor mRNA expression. The administration of morphine significantly increased both A1 and A2A receptor expression levels in the hippocampus and in the prefrontal cortex of the studied mice. The higher expression of A1 receptors was significantly reduced by SB-334867 in the hippocampus, but not in the prefrontal cortex, while the higher expression of A2A receptors was significantly reduced in the prefrontal cortex, but not in the hippocampus. The obtained results demonstrate, for the first time, a close relationship between orexin and adenosine receptors, related to SB-334867-induced changes in the expression of A1 and A2A receptors. Adenosine, as an important neuromodulator in the brain, mediates multiple pathophysiological functions mainly via adenosine receptors. A1 receptors are pre- and post-synaptically located in the whole brain, while A2A receptors are mostly presynaptically located, except for the striatum, in which A2A receptors occur post-synaptically [61]. These receptors, by their interactions with numerous neurotransmitters, such as dopamine and glutamate, take part in the regulation of synaptic transmission [62]. One of the initial studies on orexin and adenosine interactions showed that A1 receptors were located on orexin neurons in the hypothalamus [63]. Later on, it was also demonstrated that adenosine inhibited via A1 receptors orexin neurons in that area [64]. Similarly, another study showed that endogenous adenosine, acting on A1 receptors, was able to attenuate the basal excitatory synaptic transmission and inhibit long-lasting synaptic plasticity on orexin neurons [65]. The studies on the interactions between orexins and adenosine are not advanced, and the precise mechanisms, involved in expression of A1 and A2A receptors, are still not recognized. Our study demonstrates for the first time the influence of the blockade on OX-1 receptors on mRNA expression of adenosine receptors.

It is well-known that behavioral sensitization leads to some dysregulation of mesocorticolimbic pathways [9, 12, 16, 66, 67]. Dopamine is the primary neurotransmitter, involved in the rewarding activity of abused drugs [68], and dopaminergic neurons in VTA are strongly implicated in the regulation of drug-seeking behavior [69]. Moreover, the role of glutamatergic projections from the prefrontal cortex to nucleus accumbens in drug-seeking behavior is also incontestable [10]. The medial prefrontal cortex provides glutamate innervation of dopamine and GABAergic neurons in VTA [70, 71] and regulates dopamine release in the nucleus accumbens [72, 73]. Hypothalamic orexin neurons project widely throughout the brain, and a high density of anatomical orexin projections was evidenced from the hypothalamus into the VTA [74], where a large population of dopaminergic and glutamatergic receptors also occur [75, 76]. Therefore, the VTA is considered to be the major site of action for orexin in learning drug-stimulus associations [77]. For example, an infusion of SB-334867 into the VTA attenuated the acquisition of morphine-conditioned place preference in rats [78]. It was documented that orexins interacted with dopamine and glutamate neurons in the VTA. For example, neurophysiological studies revealed orexin to have augmented responses of dopamine neurons in the VTA to activation of the medial prefrontal cortex [77] and that an administration of orexin A in an in vitro study induced a certain potentiation of glutamate neurotransmission on dopamine neuron synapses within the VTA [28]. Thus, the administration of SB-334867, a selective antagonist of OX-1, confirmed in our study the role of OX-1 receptors in morphine-induced behavioral sensitization and evidenced connections of OX-1 receptors with dopamine D1 and D2 and adenosine A1 and A2A receptors in the mesolimbic areas. Although other interactions of orexin neurons within the mesolimbic system cannot be ruled out, we suggest that the interactions of orexin neurons with dopamine and glutamate neurons within VTA were of key significance for the alterations in mRNA expression of orexin, dopamine, and adenosine receptors, observed in the presented results.

In the subsequent step of our study, we investigated the expression of the markers of glial cells (GFAP and Iba-1) in morphine-induced sensitization. A growing number of evidences demonstrates that a long-term administration of abused drugs may induce higher expression levels of glial cells, what supports the role of neuroinflammatory processes in addiction and dependence [44, 45]. It was evidenced that a chronic exposure to morphine induced an increased expression of GFAP and Iba-1 in the hippocampus [79, 80] and VTA [81–83]. On the other hand, the results of the experiments, showing the expression of glial cells in the striatum, are diversified. For example, Marie-Claire et al. [84] observed an increase in GFAP expression, while Campbell et al. [85] demonstrated a reduction of Iba-1 expression. Most of the studies on glial activation are focused on the models of tolerance and dependence. The expression of glial cells in a morphine sensitization model was studied for the first time in the present study. We demonstrated that an mRNA analysis of GFAP, an important marker of astrocyte activation, showed an increase in GFAP expression in the striatum and in the prefrontal cortex, while the administration of SB-334867 reduced GFAP expression. However, GFAP expression was significantly reduced in the hippocampus of the morphine-sensitized mice, and that effect was abolished by the treatment with SB-334867. On the other hand, the expression of Iba-1, a marker of microglial activation, was significantly reduced in all the studied brain areas after the sporadic administration of morphine. Those reductions were completely restored by SB-334867. Thus, we hypothesized that a sporadic morphine administration can modulate astrocyte and reduce microglial cell expression.

Astrocytes, as an important factor of “tripartite synapse,” can contact thousands of synapses and support the functioning of neurons. They control and alter synaptic functions by modulation of the release of glutamate, purines, adenosine triphosphate (ATP), adenosine, dopamine, GABA, and others [86–92]. Microglia, as the resident macrophage cells, act as the primary and main form of active immune defense in the CNS. They maintain homeostasis in non-infected regions and promote inflammation in infected or damaged tissues [93]. Neurons can activate glial cells via neuromodulators, such as glutamate, nitric oxide, ATP, and others. Conversely, the activated glial cells affect neuronal functions, what is considered to be the pathomechanism of various disorders. Although the increased expression of glial cells in various models has repeatedly been documented, no connections between astrocytes and microglial cells have yet been recognized. For example, the activated microglia can promote astrocytes, while on the other hand, astrocytes can either stimulate or inhibit microglial activities, depending on inflammation intensity [93].

Summing up, we evidenced in the presented study that SB-334867, an OX-1 receptor antagonist, inhibited the acquisition of morphine-induced sensitization, in mice. Additionally, we showed a broad range of receptor interactions among orexin, dopamine, and adenosine, occurring in the mesolimbic system and involved in those processes. Finally, we demonstrated the role of glial cells in a morphine sensitization model. Taking into account the results of the presented study, it may be concluded that the orexin system may be a valuable tool in controlled inhibition of morphine-induced behavioral sensitization.

## Abbreviations

ATP: Adenosine triphosphate
CNS: Central nervous system
D1: Dopamine receptor type 1
D2: Dopamine receptor type 2
DMSO: Dimethyl sulphoxide
GABA: γ-Aminobutyric acid
GFAP: Glial fibrillary acidic protein
i.p.: Intraperitoneally
Iba-1: Ionized calcium-binding adapter molecule 1
OX-1: Orexin type 1
OX-2: Orexin type 2
SB-334867: 1-(2-Methyylbenzoxanzol-6-yl)-3-[1,5]naphthyridin-4-yl-urea hydrochloride
VTA: Ventral tegmental area
